# Engineering a Dimeric Caspase-9: A Re-evaluation of the Induced Proximity Model for Caspase Activation

**DOI:** 10.1371/journal.pbio.0030183

**Published:** 2005-05-10

**Authors:** Yang Chao, Eric N Shiozaki, Srinivasa M Srinivasula, Daniel J Rigotti, Robert Fairman, Yigong Shi

**Affiliations:** **1**Department of Molecular Biology, Lewis Thomas LaboratoryPrinceton University, Princeton, New JerseyUnited States of America; **2**Laboratory of Immune Cell Biology, National Cancer InstituteNational Institutes of Health, Bethesda, MarylandUnited States of America; **3**Department of Biology, Haverford CollegeHaverford, PennsylvaniaUnited States of America; University of Texas Southwestern Medical CenterUnited States of America

## Abstract

Caspases are responsible for the execution of programmed cell death (apoptosis) and must undergo proteolytic activation, in response to apoptotic stimuli, to function. The mechanism of initiator caspase activation has been generalized by the induced proximity model, which is thought to drive dimerization-mediated activation of caspases. The initiator caspase, caspase-9, exists predominantly as a monomer in solution. To examine the induced proximity model, we engineered a constitutively dimeric caspase-9 by relieving steric hindrance at the dimer interface. Crystal structure of the engineered caspase-9 closely resembles that of the wild-type (WT) caspase-9, including all relevant structural details and the asymmetric nature of two monomers. Compared to the WT caspase-9, this engineered dimer exhibits a higher level of catalytic activity in vitro and induces more efficient cell death when expressed. However, the catalytic activity of the dimeric caspase-9 is only a small fraction of that for the Apaf-1-activated caspase-9. Furthermore, in contrast to the WT caspase-9, the activity of the dimeric caspase-9 can no longer be significantly enhanced in an Apaf-1-dependent manner. These findings suggest that dimerization of caspase-9 may be qualitatively different from its activation by Apaf-1, and in conjunction with other evidence, posit an induced conformation model for the activation of initiator caspases.

## Abstract 2

Evidence from an engineered caspase-9 dimer suggests that, contrary to the prevailing hypothesis, dimerization may not be the critical step in caspase-9 activation.

## Introduction

Apoptosis plays a central role in animal development and tissue homeostasis. The essential machinery responsible for the execution of apoptosis is the caspases, a family of closely related cysteine proteases [1–6]. Caspases comprise two classes: the initiator caspases, such as caspase-8 and -9, and the effector caspases, such as caspases-3 and -7. Due to their deleterious roles, all caspases are synthesized as relatively inactive zymogens in the cell and, in response to apoptotic stimuli, undergo proteolytic activation. The activation of effector caspases is performed by the initiator caspases. The autoactivation of the initiator caspases is facilitated by other auxiliary factors. For example, the autoactivation of caspase-9 is mediated by the assembly of a heptameric complex involving Apaf-1 and cytochrome c, dubbed the apoptosome [7–9]. Once activated, caspase-9 cleaves and activates caspases-3 and -7.

The molecular mechanism for the activation of the effector caspases has been elucidated [6]. The caspase zymogen, such as procaspase-7, exists as a homodimer in solution but exhibits poor catalytic activity because its active sites exist in an unproductive conformation. The activation cleavage of procaspase-7 allows the relocation of a surface loop from one monomer to critically support the active site of the adjacent monomer, hence allowing catalysis to proceed [10,11].

In contrast, the mechanism for the activation of the initiator caspases, which generally involves the formation of an oligomeric complex [9], has remained elusive [12]. The prevailing hypothesis has been the “induced proximity” model, which summarized a set of elegant experiments [13–17] to state that caspase zymogens are autoprocessed once they are brought into proximity with each other [18]. However, this initial explanation of the induced proximity model contained little mechanistic information until it was enhanced by the proposition that the oligomeric complex (such as the apoptosome) serves to activate the monomeric initiator caspases through induced dimerization via their intrinsic dimerization interface [19–21]. Supporting this hypothesis, caspase-9 exists predominantly as a monomer in solution and exhibits a basal level of catalytic activity [19,22]. Induced proximity, interpreted as dimerization-driven activation of caspases, is perceived as a dominant model of initiator caspase activation [19–21,23].

Despite its popularity, the notion of dimerization-driven activation of initiator caspases is at odds with some well-known facts. For example, if the activation of the initiator caspases requires merely dimerization, why would the apoptosome exhibit a 7-fold symmetry? Along this line, the activation of caspase-8 requires the assembly of a multi-component death-inducing signaling complex, which has a 3-fold symmetry.

The induced proximity model states that the dimerization of caspase-9, via its intrinsic dimerization interface, is the central and only step in its activation, and the assembly of the apoptosome serves merely to facilitate the dimerization of caspase-9 [19,20,23]. Thus, caspase-9 in its dimeric form is expected to exhibit the same catalytic activity as caspase-9 when it is activated by the apoptosome. An essential experiment in assessing the correctness of this prediction is to directly compare the catalytic activity of the dimeric caspase-9 with that of the apoptosome-activated caspase-9. Unfortunately, the wild-type (WT) caspase-9 dimer defies isolation, because it does not represent a stable state in solution [22]. The only approach is to engineer a constitutive caspase-9 homodimer that will dimerize via its intrinsic dimerization interface.

In this report, we revisit the induced proximity model through the engineering of a constitutively dimeric caspase-9. We demonstrate that, despite the fact that this dimer variant exhibits a higher level of catalytic activity in vitro and induces more efficient cell death than the WT caspase-9, it is qualitatively different from the Apaf-1-activated caspase-9. Importantly, the structure of the engineered caspase-9 closely resembles that of the WT caspase-9, including all relevant structural details and the asymmetric nature of the two monomers. Our data, in conjunction with other evidence, suggest a refined model of the induced proximity hypothesis. At the center of this model is an induced conformation of the active site, hence the name induced conformation model.

## Results

### Rationale for the Design of a Dimeric Caspase-9

While all members of the caspase family share a similar overall structure, small differences in their primary sequences lead to their individuality [1,4]. An effector caspase, such as caspase-3, exists exclusively as a stable homodimer in solution. In contrast, biochemical and structural analyses revealed that dimerization of the initiator caspase-9, although possible, was energetically unfavorable [1,19,22]. To facilitate the design of a constitutively dimeric caspase-9, we examined the dimerization interface of several representative caspases.

In caspase-3, the dimerization interface is mediated primarily by two β-strands (β6 and β6′), one from each monomer (Figure 1A). Together, 63% of the buried surface area at the dimeric interface is contributed by these two centrally located β-strands and their immediate flanking residues. Consistent with a critical role in dimerization, residues on these two β-strands exhibit relatively low temperature factors. Importantly, sequence alignment among four representative caspases revealed an extremely varied section of five consecutive amino acids on strand β6, flanked on either side by stretches of conserved residues (Figure 1B). For example, the five residues Cys^264^-Ile-Val-Ser-Met^268^ in caspase-3 are replaced by Gly^402^-Cys-Phe-Asn-Phe^406^ in caspase-9, while five of the eight flanking residues are invariant between these two caspases. Compared to caspase-3, caspases-6 and -7, also known to exist as homodimers in solution, contain similarly conserved residues on the β6 strand (Figure 1B). This observation further supports a critical role of the β6 strand in dimerization and suggests a conserved pattern required for dimerization. In agreement with this analysis, previous structural investigation showed that Phe^404^ and Phe^404^′, one of the five residues on strands β6 and β6′, respectively, appear to impede the dimerization of caspase-9 by way of their incompatible side-chain configuration at the interface (Figure 1A) [19].

**Figure 1:**
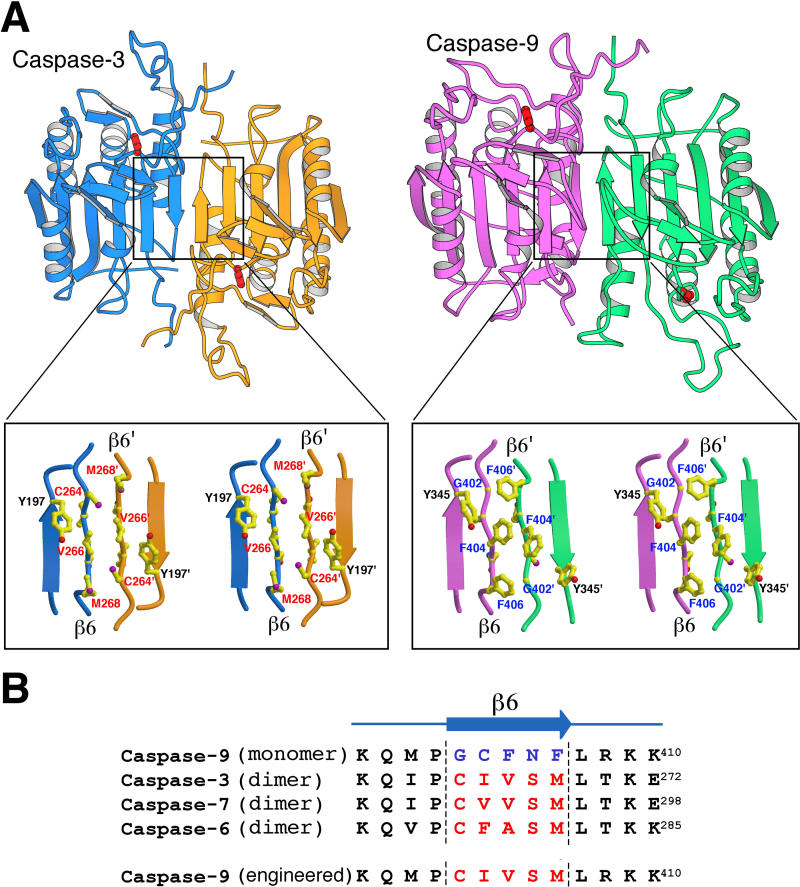
The β6 Strand Is the Major Determinant for the Dimerization of Caspases (A) Comparison of the dimerization interfaces of caspase-3 and caspase-9. Caspases-3 and -9 exist primarily as a dimer and a monomer, respectively, in solution. However, inhibitor-bound caspase-9 was crystallized in its dimeric form [19]. The overall structures of caspases-3 and -9 are similar. A close-up view of the dimerization interfaces reveals sharp variation of residues on the β6 strand between caspase-3 and -9, which likely contributes to their different propensity for dimer formation. For caspase-9, Phe^404^ on strand β6 appears to impede dimerization as it sterically clashes with Phe^404^′ of the adjacent monomer [19]. (B) A sequence alignment of the residues on and surrounding the strand β6 in four representative caspases. Similar to caspase-3, caspases-6 and -7 also exist as homodimers in solution. Residues on β6 are highlighted in blue and orange for the monomeric and dimeric caspases, respectively. Note that the variation of residues on β6 strand appears to correlate with the propensity for dimerization.

In addition to strand β6, two surface loops also appear to contribute to the dimerization of both caspases-3 and -9. Although residues from these surface loops also differ between the two caspases, they are involved in a coordinated change. For example, Asp^231^ on chain A of a caspase-3 dimer hydrogen bonds to His^234^ on chain B, while the same hydrogen bond is spatially conserved between Lys^410^ on chain A and Ser^382^ on chain B in caspase-9 [19]. Thus we concluded that these surface loops in caspase-9 play a similar role as in caspase-3 and are unlikely to be the impediment for the dimerization of caspase-9.

### The Engineered Caspase-9: A Constitutive Homodimer

The drastic difference in sequences as well as in bonding arrangements of strand β6 between caspases-3 and -9 led us to conclude that the higher propensity of caspase-3 to dimerize than caspase-9 is a direct result of the sequence variation in strand β6. To generate a potentially dimeric caspase-9, we replaced five residues in the β6 strand of caspase-9 (Gly^402^-Cys-Phe-Asn-Phe^406^) with those in caspase-3 (Cys^264^-Ile-Val-Ser-Met^268^), using standard mutagenesis methods. This engineered caspase-9 was overexpressed in bacteria and purified to homogeneity (see Materials and Methods for details).

First, we compared the oligomeric states of the engineered and WT caspase-9 using size exclusion chromatography. The elution volume for the WT protein (residues 1–416), which contains a prodomain and a flexible linker segment between the prodomain and the protease domain, corresponds to a molecular mass of approximately 60 kDa (Figure 2), slightly larger than that expected for a monomer (approximately 46 kilodaltons [kDa]). This is consistent with the fact that the prodomain and the flexible linker segment of caspase-9 may increase its hydrodynamic radius in solution. In contrast, the elution volume of the engineered caspase-9 (residues 1–416) corresponds to a molecular mass of about 120 kDa (Figure 2), approximately twice that observed for the WT protein. To better correlate elution volume with molecular mass, WT and engineered proteins were also prepared in which the prodomain and the flexible linker were removed (Δ139). In this case, the elution volumes for the WT and engineered caspase-9 (Δ139, residues 140–416) corresponded to 30 kDa and 60 kDa, respectively, consistent with a monomer and a dimer of caspase-9 (Δ139) (unpublished data).

**Figure 2:**
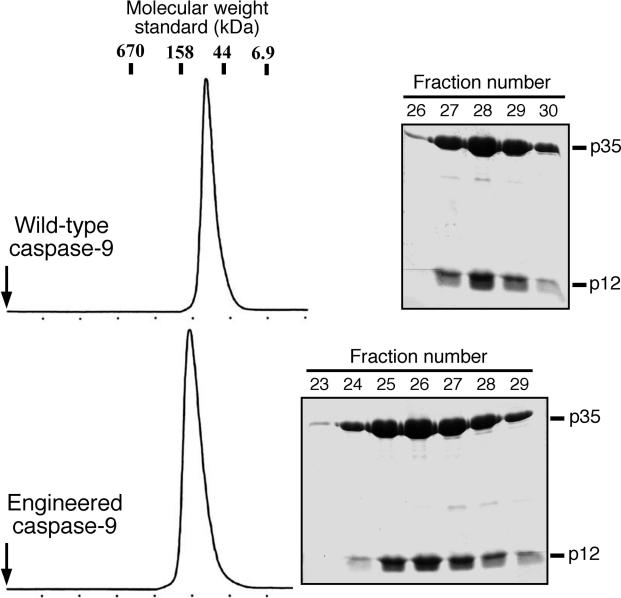
The Dimeric Caspase-9 Exists as a Monomer in Solution The apparent molecular masses for the WT and dimeric caspase-9 (full-length, residues 1–416) were analyzed by gel filtration. Relevant peak fractions were visualized by SDS-PAGE followed by Coomassie staining. The dimeric caspase-9 was eluted from the column with a molecular mass about twice that of the WT protein.

To precisely determine the molecular mass of the engineered caspase-9, we performed sedimentation equilibrium experiments using analytical ultracentrifugation. Analysis of the data for the engineered caspase-9 (residues 1–416), at three rotor speeds, reveals a molecular mass of 91,030 ± 2,100 daltons (Da) (Table 1). This is very close to the expected molecular weight of a homodimer (94,610 Da). In contrast, the WT caspase-9 exhibited a molecular mass of 50,550 ± 2,550 Da (Table 1), slightly larger than that of a monomer (47,357 Da). Data fitting indicated that the slightly larger molecular mass is not due to a monomer-dimer equilibrium. Rather, it is likely due to a small degree of higher-order nonspecific association among the monomers. Similarly, analysis of the data for the WT and engineered caspase-9 (Δ139) reveals molecular masses of 36,450 ± 3,300 and 61,490 ± 2,350 Da, respectively. These values are close to the predicted molecular masses of monomeric (31,426 Da) and dimeric (62,746 Da) caspase-9 (Δ139).

**Table 1:**
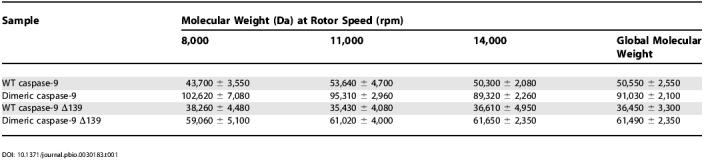
Analytical Ultracentrifugation Analysis of Caspase-9 Proteins

Thus, results derived from gel filtration and analytical ultracentrifugation analysis unequivocally show that our engineered caspase-9, which contains a five-residue replacement at the dimeric interface, exists predominantly as a homodimer in solution. For the sake of discussion, the engineered caspase-9 is hereafter referred to as the dimeric caspase-9.

### Structure of the Dimeric Caspase-9

One important assumption from our engineering effort is that the replacement of five amino acids in strand β6 would not significantly alter the local structure of the dimeric caspase-9 surrounding strand β6, as compared to the WT caspase-9. Although this assumption is supported by the highly conserved nature of caspase structures, it must be proved experimentally. To obtain a structure of the dimeric caspase-9, we mounted a vigorous crystallization effort. However, the enhanced enzymatic activity (see below) of the dimeric caspase-9 led to time-dependent degradation of the protein under a variety of conditions (unpublished data) that impeded crystallization. To solve this problem, we mutated the catalytic residue Cys^287^ to Ser and were able to crystallize this catalytically inactive form of the dimeric caspase-9. The structure was determined at 2.8 Å (Figure 3A and Table 2).

**Figure 3:**
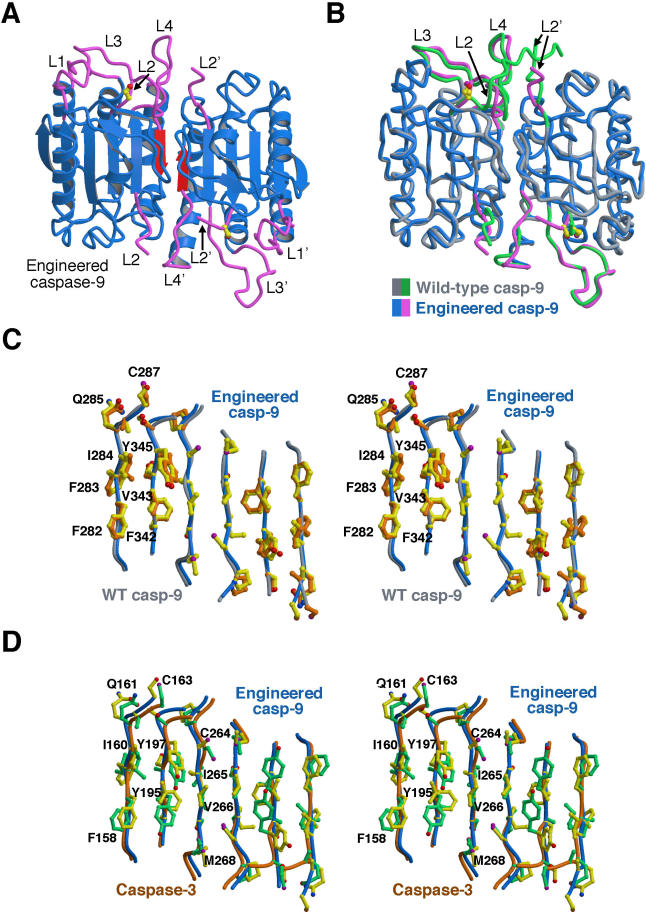
The Dimeric Caspase-9 Closely Resembles the WT Caspase-9 (A) Overall structure of the dimeric caspase-9 (C287S). The structural core is shown in blue; the solvent-exposed active site loops are shown in magenta. The β6 and β6′ strands are highlighted in red. Note the asymmetry of the dimer. (B) Structural superposition of the dimeric caspase-9 (blue and magenta) and the WT caspase-9 (grey and green). The only significant structural difference is in the solvent-exposed active site loops, which is due to the inactive nature of the dimeric caspase-9 (C287S). (C) A stereo comparison of the region surrounding strands β6 and β6′ in WT and dimeric (engineered) caspase-9. The side chains of the WT and dimeric caspase-9 are shown in orange and yellow, respectively. To avoid congestion in the graphic, residues from only one caspase-9 monomer are labeled. The Cys^287^ in the upper left corner is the catalytic residue in the active site of the asymmetric caspase-9. (D) A stereo comparison of the region surrounding strands β6 and β6′ in caspase-3 and dimeric (engineered) caspase-9. The side chains of the caspase-3 and dimeric caspase-9 are shown in green and yellow, respectively. The labels refer to amino acids in caspase-3.

**Table 2:**
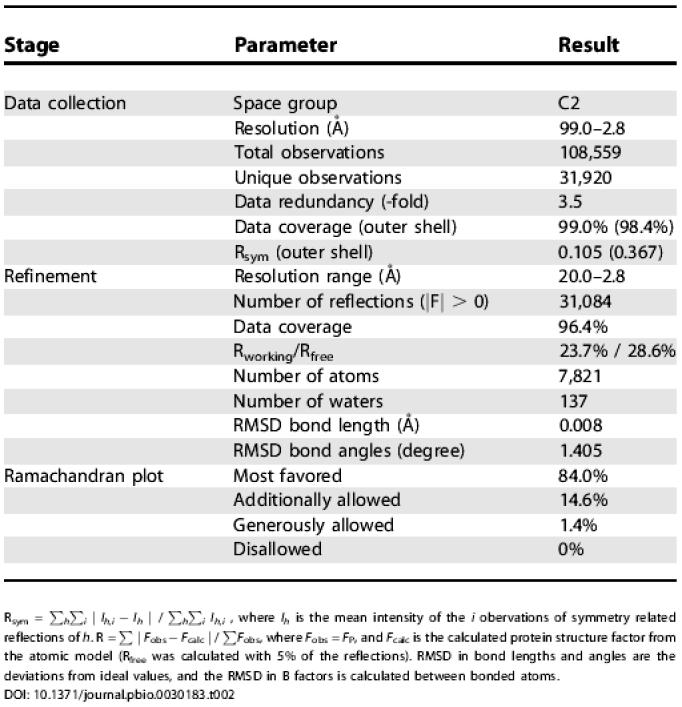
Data Collection and Statistics from Crystallographic Analysis

R_sym_ = *∑_h_∑_i_* | *I_h,i_* − *I_h_* | / *∑_h_∑_i_ I_h,i_* , where *I_h_* is the mean intensity of the *i* obervations of symmetry related reflections of *h*. R = ∑ | *F*_obs_ − *F*_calc_ | / ∑*F*_obs_, where *F*_obs_ = *F*_P_, and *F*_calc_ is the calculated protein structure factor from the atomic model (R_free_ was calculated with 5% of the reflections). RMSD in bond lengths and angles are the deviations from ideal values, and the RMSD in B factors is calculated between bonded atoms.

This structure offers a number of interesting findings, all of which support the assumption that the five-residue replacement does not alter the local structure of caspase-9 surrounding strand β6. First, the dimeric caspase-9 exists as an asymmetric homodimer in an identical manner to the WT caspase-9 [19]. In fact, the dimeric caspase-9 was crystallized in the same space group as the WT caspase-9 with essentially identical unit cell parameters. Just like the WT caspase-9, there are two molecules of the dimeric caspase-9 in each asymmetric unit, and the crystal packing interaction is identical. This observation also indicates that Phe^404^ is not a contributing reason for the observed asymmetry in caspase-9 as previously suggested [19], as Phe^404^ is replaced by Val in the dimeric caspase-9. Rather, the asymmetry appears to be an intrinsic property of caspase-9 determined by other sequence elements. It should be pointed out that, in theory, crystal-packing interactions could be the reason for both WT and dimeric caspase-9 to adopt a similar conformation in the crystals. However, such packing interactions are usually extremely weak compared to the forces that govern protein stability and are generally unable to perturb any well-formed structural elements.

Second, despite being an inactive zymogen, the dimeric caspase-9 is nearly identical to the WT caspase-9 for the core structural elements, with a root mean square deviation (RMSD) of 0.4 Å over 380 aligned Cα atoms (Figure 3B). The only significant structural difference occurs at the solvent-exposed surface loops L2, L4, L2′, and L4′ (Figure 3B). These differences reflect the active and inactive nature of the WT caspase-9 and dimeric caspase-9, respectively. As is the case for all other active caspases [1], the conformation of these four loops in the inhibitor-bound dimeric caspase-9 is expected to be identical to that of the inhibitor-bound WT caspase-9.

Third and most importantly, the conformation of the amino acids surrounding strands β6 and β6′ in the dimeric caspase-9 remains extremely similar or identical to that in the WT caspase-9 (Figure 3C). For example, all amino acids in the neighboring strands β5 and β5′ in the dimeric caspase-9 retain the same side chain rotamer conformation as in the WT caspase-9 (Figure 3C). There is no significant conformational change in any part of the local structure. Thus we conclude that the impact of the five-residue replacement is naturally absorbed without any significant structural rearrangement, and that the dimeric caspase-9 faithfully mimics the dimerized state of the WT caspase-9.

We also compared the dimeric caspase-9 to caspase-3 (Figure 3D). The ten Cα atoms on strands β6 and β6′ of the dimeric caspase-9 superimpose very well with the corresponding atoms in caspase-3, exhibiting an overall RMSD of 0.46 Å (Figure 3D). The surrounding residues, however, show significant deviation in both main chain and side chain atoms (Figure 3D). Structural alignment of the entire dimeric caspase-9 and caspase-3 results in an RMSD of 1.74 Å over 396 Cα atoms.

### The Dimeric Caspase-9: More Potent than the Wild Type

Previous structural studies on procaspase-7 demonstrate that the active site conformation in one monomer exists in an unproductive conformation until it is supported by the critical loop L2′ from the adjacent monomer [10,11]. This conclusion is thought to be generally applicable to other caspases [1]. Therefore, the dimeric caspase-9 is predicted to exhibit a higher level of catalytic activity than its monomeric WT counterpart, because the dimeric caspase-9 is poised to provide the L2′ loop. To examine this hypothesis, we reconstituted an in vitro assay in which the ability of caspase-9 to cleave its physiological substrate, procaspase-3 (C163A), was measured in a time-course experiment (Figure 4A). As anticipated, both the full-length and the Δ139 dimeric caspase-9 (unpublished data) exhibited a significantly higher activity than their WT counterparts. To accurately determine the differences in activity, we repeated these experiments using the fluorescent substrate specific for caspase-9, LEHD-AFC (Figure 4B). These results revealed that the full-length and the Δ139 dimeric caspase-9 were approximately 2-fold and 5-fold more active than their WT counterparts, respectively.

**Figure 4:**
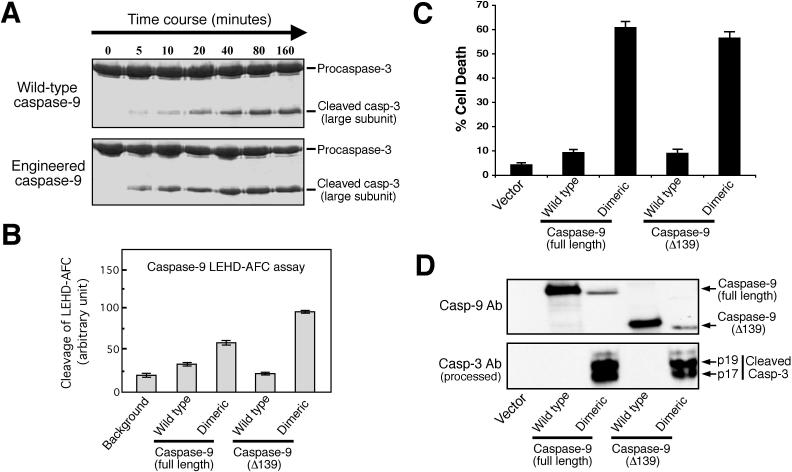
The Dimeric Caspase-9 Exhibits Higher Catalytic Activity and Stronger Cell-Killing Activity than the WT Protein (A) A time course experiment of caspase-9 activity using procaspase-3 (C163A) as the substrate revealed that the dimeric caspase-9 (residues 1–416) exhibited an approximately 5-fold higher level of catalytic activity than the monomeric WT caspase-9. The concentrations were: WT and engineered caspase-9, 0.5 μM; procaspase-3 (C163A), 33 μM. (B) Comparison of catalytic activity for the WT and dimeric caspase-9 using fluorescent substrate LEHD-AFC. Both the full-length and Δ139 dimeric caspase-9 displayed higher activities than their WT counterparts. (C) The dimeric caspase-9 induced apoptosis more effectively than the WT protein. Mammalian expression vector pcDNA3 constructs encoding WT or dimeric caspase-9 were transfected into HeLa or 293 cells. The extent of cell death induced by each construct was examined. (D) The dimeric but not the WT caspase-9 promoted the activation of caspase-3. The expression of caspase-9 variants (upper panel) and the processing of caspase-3 (lower panel) from the cell extracts were detected by antibodies against caspase-9 and -3, respectively. No endogenous caspase-9 band was visible in the vector-transfected cells at lower exposure.

Increased catalytic activity for the dimeric caspase-9 is predicted to correlate with stronger ability in promoting apoptosis. To test this prediction, the WT and dimeric caspase-9 were expressed in HeLa and HEK 293 cells; indeed, the dimeric caspase-9 induced extensive cell death in both HeLa (Figure 4C) and HEK 293 cells (unpublished data), whereas overexpression of the WT caspase-9 induced little apoptosis. The induction and extent of apoptosis were confirmed by the processing of caspase-3 in cells expressing the dimeric but not the WT caspase-9 (Figure 4D). Despite its strong ability in inducing apoptosis, the dimeric caspase-9 was expressed at a much lower level than the WT enzyme (Figure 4D). This likely reflects the cytotoxic effect of the dimeric caspase-9 once expressed, or a shorter half-life of the processed dimeric caspase-9. The activation of caspase-3 and induction of apoptosis by the dimeric caspase-9 no longer require the caspase-9-activating factor Apaf-1, because the dimeric caspase-9 (Δ139), which lacks the Apaf-1-binding domain, CARD, was still able to induce efficient cell death and caspase-3 activation (Figure 4C and 4D). These data confirm that the dimeric caspase-9 is active in mammalian cells and that it induces apoptosis by activating caspase-3.

### The Dimeric Caspase-9 Is Qualitatively Different from the Apaf-1-Activated Caspase-9

If induced proximity, interpreted as dimerization-driven activation of caspases [23], models the correct mechanism for the activation of caspase-9, then the dimeric caspase-9 should exhibit a similar level of catalytic activity to the apoptosome-activated caspase-9. To examine this scenario, we reconstituted an apoptosome-activated caspase-9 assay using an Apaf-1 fragment (residues 1–570) that was known to activate caspase-9 in a cytochrome c-independent manner [16,24,25]. Compared to the dimeric caspase-9, the Apaf-1-activated WT caspase-9 exhibited an approximately 35-fold higher activity using LEHD-AFC as the substrate (Figure 5A). We also confirmed this result by performing a time course experiment (Figure 5B), and obtained the same conclusion using procaspase-3 (C163A) as the substrate (unpublished data). These results suggest that the dimerization of caspase-9 may be qualitatively different from the way in which Apaf-1 activates caspase-9.

**Figure 5:**
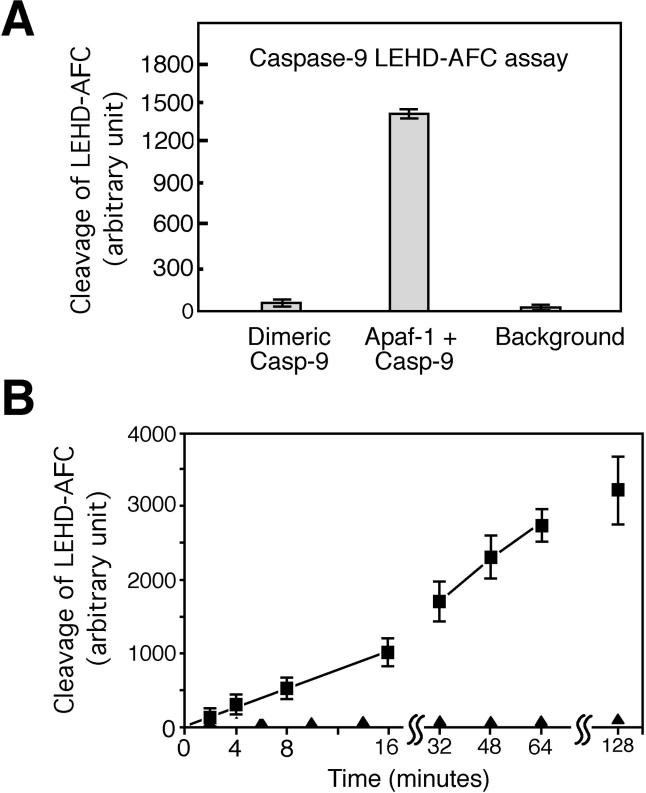
The Dimeric Caspase-9 Exhibits a Much Lower Activity Than the Apaf-1-Activated WT Caspase-9 (A) Using fluorescent substrate LEHD-AFC, the catalytic activity of the Apaf-1-activated WT caspase-9 was approximately 35-fold greater than that of the dimeric caspase-9. (B) A time course comparison of the catalytic activities between the Apaf-1-activated WT caspase-9 and the dimeric caspase-9. WT and dimeric caspase-9, 0.5 μM; Apaf-1 (residues 1–570), 2 μM; LEHD-AFC, 200 μM.

Next, we examined whether the dimeric caspase-9 can be activated by Apaf-1 the same way as the WT caspase-9. Using procaspase-3 (C163A) as the substrate, the catalytic activity of the dimeric caspase-9 does not change appreciably in the presence of increasing amounts of Apaf-1 (Figure 6A, lanes 5–8). In contrast, the WT caspase-9 was activated by Apaf-1 in a concentration-dependent manner (Figure 6A, lanes 1–4). Subsequently, in the presence of Apaf-1, the catalytic activity of WT caspase-9 significantly exceeded that of the dimeric caspase-9 (Figure 6B).

**Figure 6:**
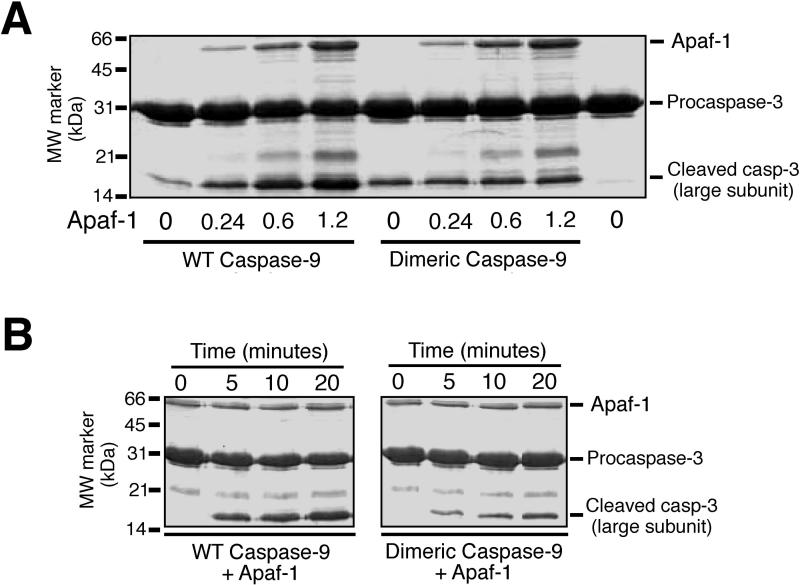
The Dimeric Caspase-9 Can No Longer Be Activated the Same Way as the WT Caspase-9 (A) Increasing amounts of Apaf-1 (residues 1–570) led to a corresponding increase in the catalytic activity of the WT caspase-9, but had no effect on the activity of the dimeric caspase-9. WT and dimeric caspase-9, 0.5 μM; procaspase-3 (C163A), 33 μM; Apaf-1, 0.24, 0.6, and 1.2 μM. (B) A time course of procaspase-3 cleavage by the WT and dimeric caspase-9 in the presence of Apaf-1. WT and dimeric caspase-9, 0.3 μM; procaspase-3 (C163A), 33 μM; Apaf-1, 0.3 μM.

## Discussion

Although induced proximity through high levels of protein expression can lead to caspase activation, it remains questionable whether this is indeed how the initiator caspases are activated under physiological conditions [12]. In fact, much of the evidence supporting the induced proximity model can also be used to argue against it. For example, effector caspases can be autoactivated through induced proximity when overexpressed in bacteria; yet in mammalian cells the endogenous effector caspases are activated by the initiator caspases. Thus, merely showing the processing of caspases due to forced oligomerization does not constitute strong support of the induced proximity model and does not explain how initiator caspases are activated in cells. More importantly, the initial proposition of the induced proximity model gives little or no consideration to the most fundamental aspect of cellular biochemistry: specificity, the specific protein-protein interactions that are required for the precise positioning and activation of the initiator caspases.

It should be noted that the concept of caspase activation is fundamentally different between the effector and the initiator caspases. The effector caspases, including the zymogens, exist as constitutive homodimers in solution. Their activation requires an interdomain cleavage that facilitates the formation of a productive active site conformation. However, for the initiator caspases, activation has a quite different meaning. For example, the processed caspase-9, similar to the unprocessed procaspase-9, is only marginally active [26,27]; the primary function of the apoptosome is to up-regulate caspase-9 activity rather than to facilitate its autoprocessing [26,28]. In fact, the unprocessed caspase-9 can be activated to the same level by the apoptosome as the processed caspase-9 [27]. Thus, the activation of caspase-9 is reflected by its association with the apoptosome and not by the interdomain cleavage. It remains to be seen whether caspase-9 activation represents an isolated example or a general theme among the initiator caspases.

The reported experimental evidence supporting the initial induced proximity model employed means to facilitate the oligomerization of caspases using heterologous domains that dimerize constitutively or upon binding to ligands [13–15,17]. The design of these experiments allowed caspases to be brought close to each other via their attached dimerization domains; however, it was not shown that the caspases themselves had dimerized via their intrinsic dimerization interface. The initial induced proximity model was given greater mechanistic meaning when it was explicitly proposed that dimerization via the intrinsic dimerization interface of initiator caspases drives their activation [19]. This improved interpretation of the induced proximity model impinges upon specific protein conformation and was perceived as the dominant model to explain the mechanism of initiator caspase activation [20,21,23].

To validate this induced proximity model, the activity of the dimerized caspase, such as caspase-9, must be compared with that of the apoptosome-activated caspase-9. However, a WT caspase-9 homodimer cannot be isolated because caspase-9 exists predominantly as a monomer in solution [22]. If there is any tendency for the WT caspase-9 to dimerize, the kinetics must be exceedingly fast, since dimeric caspase-9 has eluded detection by all biochemical means in our hands. It should also not noted that, although a stable caspase-9 homodimer was reported to exist in solution [19], rigorous effort in several laboratories, including ours, to reproduce this result have not been successful.

Using protein engineering, we generated a stable caspase-9 homodimer by changing residues exclusively at the dimerization interface. It should be noted that our dimeric caspase-9 is different from other heterologous caspase constructs reported in the literature, in which the caspases were fused to heterologous dimerization domains [13–17]. Whether the caspases can dimerize via their intrinsic dimerization interface in those circumstances remains undetermined. In contrast, our design relies on the assumptions that the engineered caspase-9 would dimerize via its intrinsic dimerization interface and would closely resemble the WT protein except at the buried dimerization interface. Importantly, these assumptions have been proved correct by our structural analysis of the dimeric caspase-9. However, the engineered dimeric caspase-9 exhibits a catalytic activity that is only a small fraction of that of the WT caspase-9 activated by Apaf-1. The discrepancy in activity suggests that dimerization of caspase-9 may be qualitatively different from the Apaf-1-mediated activation of caspase-9 and is unlikely to be responsible for the activation of caspase-9 in cells.

Interestingly, the dimeric caspase-9 exhibits an activity that is only 2- to 5-fold higher than that of the WT caspase-9 (see Figure 4B). This observation exactly argues against the prevailing hypothesis that dimerization drives activation of caspase-9, because if dimerization of caspase-9 were the mechanism for its activation, the dimeric caspase-9 should exhibit a much high level of activity—similar to that of the apoptosome-activated caspase-9. This analysis is further supported by our structural observation, which reveals that the five-residue mutation at the interface of the dimeric caspase-9 does not result in any significant conformational changes in the local structure surrounding strands β6 and β6′.

If dimerization of caspase-9 is not the major mechanism for its activation, then how is caspase-9 activated by the apoptosome? We note that the Apaf-1-mediated apoptosome has a 7-fold symmetry. The activation of another initiator caspase, caspase-8, is facilitated by the death-inducing signaling complex, which involves a homotrimeric (3-fold symmetry) assembly of the death receptor and other associated factors. One possibility is that the apoptosome may directly activate monomeric caspase-9 through modification of its active site conformation [8,12]. An alternative model is that the apoptosome assembles the dimeric caspase-9 into a higher-order complex, which results in the modification of the active site conformation for an enhanced activity [12]. In addition, it is possible that the dimerized caspase-9 in the context of the apoptosome exhibits a perturbed interface relative to the crystallographically observed interface, which may greatly facilitate the catalytic activity of caspase-9. Although available data are insufficient to differentiate among these models, exquisite conformational change of caspase-9 must be induced upon binding to the apoptosome [29]. This conformational change, most likely at the level of active site conformation, is the prerequisite for the activation of caspase-9. We propose that this induced conformation model is the mechanism for the activation of initiator caspases.

The induced conformation model for the activation of initiator caspases is different from the dimerization-driven induced proximity model, but these two models may not be mutually exclusive. In some cases, they emphasize different aspects, and initiator caspases may exist in several distinct classes. For example, for some initiator caspases, dimerization might be sufficient for inducing the correct conformation needed for its activation. In this case, the two models are in agreement with each other. However, for caspase-9, dimerization itself is unlikely to be the sole mechanism of activation. Finally, regardless of the semantics, the activation of any initiator caspase must require the formation of a productive active site conformation.

## Materials and Methods

### 

#### Protein preparation.

All constructs were generated using a standard PCR-based cloning strategy, and the identities of individual clones were verified through double-stranded plasmid sequencing. All caspase-9 constructs were expressed from the vector pET-21b in the Escherichia coli strain BL21(DE3). The protein was purified using a Ni-NTA (Qiagen, Valencia, California, United States) column, and further fractionated by anion-exchange (Source-15Q, Pharmacia, Uppsala, Sweden) and gel-filtration chromatography (Superdex-200, Pharmacia). Caspase-3 (C163A) and Apaf-1 (1–570) were overexpressed and purified as described [22].

#### Crystallization and data collection.

Crystals were grown by the hanging-drop vapor-diffusion method by mixing dimeric caspase-9 (residues 140–416, C287S) with an equal volume of reservoir solution containing 100 mM MES (pH 6.0), 5% (w/v) PEG 5000 monomethyl ether, and 3% tacsimate. Crystals appeared overnight and grew to a typical size of 0.1 × 0.5 × 0.5 mm^3^ in 3 d. The crystals were in the C2 space group, with the cell parameters a = 144.6 Å, b = 79.0 Å, c = 125.9 Å, and β = 112.5 degrees. There are two complete molecules of dimeric caspase-9 in each asymmetric unit. Diffraction data were collected using an R-AXISIV imaging plate detector mounted on a Rigaku 200HB generator (Rigaku, Tokyo, Japan). For data collection, crystals were equilibrated in a cryoprotectant buffer containing well buffer plus 20% glycerol, and were flash frozen in a −170 °C nitrogen stream. All datasets were processed using the software Denzo and Scalepack [30].

#### Structure determination of the dimeric caspase-9.

The structure was determined by molecular replacement, using the software AmoRe [31]. The atomic coordinates of caspase-9 [19] were used for rotational search against a 15−3.2 Å dataset. The top solutions from the rotational search were individually used for a subsequent translational search, which yielded the correct solutions with high correlation factors. A model was built using the program O [32] and refined using CNS [33]. The final refined atomic model for the dimeric caspase-9 contains residues 58–196 and 212–303, and 111 ordered water molecules at 2.6 Å resolution. The final refined atomic model contains residues 140–288 and 335–416 for chains A and C, and 140–289 and 337–416 for chains B and D, respectively.

#### Caspase-9 assay.

The reaction was performed at 37 °C under the following buffer conditions: 25 mM HEPES (pH 7.5), 100 mM KCl, 4 mM MgCl_2_, and 2 mM dithiothreitol (DTT). The substrate (procaspase-3 [C163A]) concentration was approximately 33 μM. Caspase-9 variants were diluted to the same concentration (0.3 or 0.5 μM) with the assay buffer. Reactions were stopped with the addition of an equivolume of 2× SDS loading buffer and boiled for 3 min. The samples were size-fractionated by SDS-PAGE, and the results were visualized by Coomassie staining. The assays using the fluorogenic substrate Ac-LEHD-AFC were performed under similar conditions, where the substrate concentration was 200 μM. Reactions were stopped by placing the samples on ice. Fluorescence emission at 440 nm was measured using an excitation wavelength of 380 nm.

#### Analytical ultracentrifugation**

Protein samples were prepared in 25 mM HEPES (pH 7.5), 100 mM NaCl, and 0.5 mm DTT. Protein loading concentrations were all 20 μM except for caspase-9 (Δ139) which was studied at 10 μM. All sedimentation equilibrium experiments were carried out at 4 °C using a Beckman Optima XL-A analytical ultracentrifuge (Beckman Instruments, Fullerton, California, United States) equipped with an An60 Ti rotor and six-channel, 12 mm path length, charcoal-filled Epon centerpieces and quartz windows. Data were collected at three rotor speeds (8,000, 11,000, and 14,000 rpm) and represent the average of 20 scans using a scan step-size of 0.001 cm. Partial specific volumes and solution density were calculated using the Sednterp program (www.bbri.org/RASMB/rasmb.html). Data were analyzed using the WinNONLIN program from the Analytical Ultracentrifugation Facility at the University of Connecticut (Storrs, Connecticut, United States).

#### Apoptosis assays.

The ability of caspase-9 variants to induce apoptosis was assayed as described earlier [16]. Human HeLa cells or HEK 293 cells were transfected in 12-well plates (10^5^ cells/well) with 0.3 μg of pEGFP-N1 reporter plasmid (Clontech, Palo Alto, California, United States) and 1.0 μg of vector, or a construct encoding WT or dimeric caspase-9 using the LipofectAMINE (Invitrogen, Carlsbad, California, United States) as per the manufacturer's recommendations. After 24 h of transfection normal and apoptotic GFP-expressing cells were counted using fluorescence microscopy. The percentage of apoptotic cells in each experiment was expressed as the mean percentage of apoptotic cells as a fraction of the total number of GFP-expressing cells. Apoptosis in the cells transfected with caspase-9 constructs was further confirmed by immunoblotting these cellular extracts with antibodies (Cell Signaling Technology, Beverly, Massachusetts, United States) raised against caspase-3 and caspase-9, respectively. The data represent the average of four independent experiments.

## Abbreviations

Da: dalton(s)
kDa: kilodalton(s)
RMSD: root mean square deviation
WT: wild-type
